# Prevalence of human pegivirus-1 and sequence variability of its E2 glycoprotein estimated from screening donors of fetal stem cell-containing material

**DOI:** 10.1186/s12985-017-0837-y

**Published:** 2017-08-31

**Authors:** Yakov Vitrenko, Iryna Kostenko, Kateryna Kulebyakina, Khrystyna Sorochynska

**Affiliations:** Cell Therapy Center Emcell, Kyiv, Ukraine

**Keywords:** Pegivirus, HPgV-1, Fetal stem cells, E2 glycoprotein

## Abstract

**Background:**

Human pegivirus-1 (HPgV-1) is a member of the *Flaviviridae* family whose genomic organization and mode of cellular entry is similar to that of hepatitis C virus (HCV). The E2 glycoprotein of HPgV-1 is the principle mediator in the virus-cell interaction and as such harbors most of HPgV-1’s antigenic determinants. HPgV-1 persists in blood cell precursors which are increasingly used for cell therapy.

**Methods:**

We studied HPgV-1 prevalence in a large cohort of females donating fetal tissues for clinical use. PCR was used for screening and estimation of viral load in viremic plasma and fetal samples. Sequence analysis was performed for portions of the 5′-untranslated and E2 regions of HPgV-1 purified from donor plasmas. Sequencing was followed by phylogenetic analysis.

**Results:**

HPgV-1 was revealed in 13.7% of plasmas, 5.0% of fetal tissues, 5.4% of chorions, exceeding the prevalence of HCV in these types of samples. Transmission of HPgV-1 occurred in 25.8% of traceable mother-chorion-fetal tissues triads. For HPgV-1-positive donors, a high viral load in plasma appears to be a prerequisite for transmission. However, about one third of fetal samples acquired infection from non-viremic individuals. Sequencing of 5′-untranslated region placed most HPgV-1 samples to genotype 2a. At the same time, a portion of E2 sequence provided a much weaker support for this grouping apparently due to a higher variability. Polymorphisms were detected in important structural and antigenic motifs of E2.

**Conclusion:**

HPgV-1 is efficiently transmitted to fetus at early embryonic stages. A high variability in E2 may pose a risk of generation of pathogenic subtypes. Although HPgV-1 is considered benign and no longer tested mandatorily in blood banks, the virus may have adversary effects at target niches if delivered with infected graft upon cell transplantation. This argues for the necessity of HPgV-1 testing of cell samples aimed for clinical use.

## Background

Human pegivirus was identified in 1995-1996 in a search for the aetiological agent of hepatitis in patients tested negative for known hepatitis viruses [1, 2]. The virus was initially named GBV-C/HGV. However, this name was later abandoned after numerous unsuccessful attempts to establish a reliable association with liver disease. The virus was assigned a new name, “pegivirus” [3], which now designates the corresponding genus [4]. The genomic organization of pegivirus is similar to that of HCV featuring a 9.4-kb RNA genome (single-stranded, positive-sense), 5′- and 3′-untranslated regions (UTR) and an open reading frame encoding for polyprotein subsequently cleaved by proteases to produce functional and structural proteins. The most studied of them are the E1 and E2 glycoproteins which facilitate viral entry and harbor the majority of antigenic determinants [5]. In contrast to HCV, pegivirus does not seem to have an ORF encoding for a distinguishable core protein [6]. Pegivirus genome contains less variability than HCV, particularly in E2 region [7, 8]. Nevertheless phylogenetic studies were able to outline six genotypes [9–11] with another one suggested recently [12].

HPgV-1 has gained elevated attention after demonstration that coinfection of HIV patients with HPgV-1 results in higher CD4+ counts, increases survival and thus could be regarded as a favorable prognostic factor [13]. HPgV-1 genotypes appear to perform unequally in the inhibition of HIV cell entry [14, 15]. Numerous studies suggested the leading role for E2 in the HPgV-1-HIV antagonism [16]. Particularly, as a membrane-targeting protein, E2 can perturb HIV gag assembly on the plasma membrane [17]. Of interest, membrane fusion and HIV interaction rely on motifs that also shape up E2 immunoreactivity [18–20].

HPgV-1 can establish persistent human infection and is found in one to 19% of healthy donors depending on their socioeconomic status and lifestyle [5]. Mother-to-infant transmission occurs more frequently for HPgV-1 than HCV, and mother’s viremia appears to be the determining factor [21–23]. Because of the lack of evident disease association [24], pegivirus has not been included in the list of infectious agents whose screening is mandatory for blood banks [25]. However, HPgV-1 was suggested to be linked to non-Hodgkin’s lymphoma, perhaps by affecting immune regulation [26]. Interestingly, HPgV-1 replication sites were shown to reside in the bone marrow, spleen [27, 28] and peripheral blood mononuclear cells [29]. In view of this, a hematopoietic stem cell precursor was hypothesized to be the primary target of HPgV-1 infections [30]. However, no stem cell infection by HPgV-1 has yet been reported in culture and clinics.

Hematopoietic stem cells (HSC) are considered to be the principle curing component in many cell therapy applications [31]. At the fetal age of less than 12 weeks, liver and spleen contain a high amount of HSC [32] and hence offer some advantages for regenerative cell medicine [32–36]. Here we report the data on HPgV-1 prevalence from the routine pathogen screening implemented at our Center. In supplement to previous reports on mother-to-infant transmission [21–23], we focus on the establishment of HPgV-1 infection at early fetal stages. To estimate genetic variation of HPgV-1 in donors of fetal stem cell-containing material, we sequenced 5′-UTR and E2 regions and noted a high variability in the latter. Possible consequences of HPgV-1 delivery to the sites targeted by stem-cell therapy are discussed.

## Methods

### Samples

Plasma and fetal tissue samples were harvested after elective termination of pregnancy at the site of surgery. A vacuum-assisted procedure was used. Each donor signed informed consent for research and clinical use of donated samples. Activity of the Emcell Cell Therapy Center is covered by State licenses, ethic approval forms and certificates that can be found at www.emcell.com. The Center disposes of a large collection of stem cell- containing samples for clinical needs. Screening results from a subset of these samples served as the basis of this study.

Fetal samples were processed as described earlier [33]. Briefly, aborted fetuses of 6-12 weeks of age were transported in a sterile transport medium made of Dulbecco-modified Eagle’s medium (DMEM) without L-glutamine with gentamicin (100 mg/ml; Thermo Fisher Scientific, Waltham, MA, USA). Fetuses were washed three times in Hank’s balanced salt solution (HBSS) without calcium and magnesium (Sigma-Aldrich, St. Louis, MO, USA) and divided into organs. Chorionic connective tissue was separated from chorionic villi and then processed as a separate sample hereafter referred to as “chorion”. Whole fetal organs and chorions were washed again three times. The efficiency of this washing procedure for removing surface-bound microbial agents was demonstrated earlier [37] [38]. Fetal organs and chorions were homogenized mechanically in HBSS without calcium and magnesium. Cell suspension was filtered through a 100-μm filter (Becton-Dickinson, Franklin Lakes, NJ, USA) and cryopreserved in the presence of 5% dimethyl sulfoxide (DMSO; Sigma-Aldrich) in HBSS with the use of an ICE Cube 14 freezer (Sy-LAB Geräte GmbH, Neupurkersdorf, Austria). Samples were stored in liquid nitrogen.

Donor’s blood was collected in a BD Vacutainer® Barricor™ Plasma Blood Collection Tube (Becton-Dickinson) and, no later than in 4 h, centrifuged at 800 rpm for 10 min. Collected plasma was in most cases immediately passed to screening for infectious agents, among which were HPgV-1 and HCV. In parallel, an aliquote of plasma was nitrogen-frozen and re-used only once in case if additional tests had been appointed.

### Polymerase chain reaction (PCR) for screening and sequencing

Viral RNA was isolated using a Nucleospin Virus Dx kit (Macherey-Nagel, Duren, Germany) from an aliquote of fetal cell suspension containing about 10^4^-10^5^ cells in 300 μl of HBSS or 300 μl of plasma. Routine screening of samples was performed using Amplisens HGV and HCV PCR kits (Interlabservice, Moscow, Russia) in which reverse transcription (RT) and amplification are combined in a single step. To exclude cross-contamination, batches of 10 to 20 clinical samples were tested alongside with a mock control sample that passed through the RNA preparation step. PCR results were detected by Taqman probe fluorescence in a CFX96 real-time PCR system (Biorad, Hercules, CA, USA). The threshold of amplification *Cq* was determined as a PCR cycle at which the amplification kinetics exceeds the level of 50 relative fluorescence units (RFU). A two-tailored Student’s *t -*test was used to estimate the significance of differences between mean *Cq* values. Viral load was estimated using a standard curve of the dependence of *Cq* on log concentration of viral RNA. To plot this curve, we ran RT-PCR with dilutions of positive control samples of known HCV or HPgV-1 RNA concentration (copies/ml) supplied with the kits. Dilutions and calculations were done as per manufacturer’s manual.

For sequencing, reverse transcription was performed using Reverta (Interlabservice, Moscow, Russia) with random hexamer primers. The final cDNA volume was 20 μl of which 5 μl were used for each PCR. PCR was performed using SsoAdvanced™ Universal SYBR® Green Supermix (Biorad, Hercules, CA, USA) and previously published primer sets for 5′-UTR (forward, 5′- CAGGGTTGGTAGGTCGTAAATCC-3′; reverse, 5′- CCTATTGGTCAAGAGAGACA-3′ [39]) and a E2 fragment (forward, 5′- GCCTCHGCCAGCTTCATCAGRTA -3′; reverse, 5′- GCCASYTGYACCATAGCYGC-3′ [9]). The PCR program was: pre-denaturation, 95 °C for 5 min; amplification, 35 cycles of 95 °C for 10 s, 60 °C for 15 s, 72 °C for 45 s; one cycle of 72 °C for 5 min. We then continued only with samples producing (i) a typical sigmoid PCR curve with SYBR Green fluorescence reaching above 400 RFU and (ii) a melting curve featuring a distinct Tm peak at above 85 °C in the CFX96 real-time PCR system. Target PCR products were agarose gel-purified and sequenced at commercial facilities using the same primers as for PCR.

### Nucleotide sequence analysis

The quality of sequences was visually inspected and ambiguous bases were corrected using the Chromas trace viewer (Technelysium, South Brisbane, Australia). MEGA5.2 freeware [40] was used for sequence alignment (the ClustalW algorithm with default parameters), estimating evolutionary distance and inferring phylogenetic trees (the Neighbor-Joining algorithm, the Tamura-Nei model including both transition and transversions, partial deletion of positions with 80% of sequence coverage). The credibility of grouping was assessed by bootstrapping, a statistical method based on numerous resampling of each nucleotide position in the alignment and calculating the probability of obtaining the same grouping. In our analysis, 1000 bootstrap replicates were used. The following sequences were used as references for alignument (denoted by their GenBank accession numbers): U36380 for genotype 1, D90600 and AF104403 for genotype 2a, U63715 for genotype 2b, AB003288 for genotype 3, AB018667 for genotype 4, AY949771 for genotype 5, and AB003292 for genotype 6 [9] [39] [41].

### Amino acid sequence analysis

MEGA5.2 was used to align translated nucleotide sequences and count the number of synonymous and nonsynonymous substitutions per site (the ClustalW algorithm with default parameters). Shannon entropy profile was constructed with help of the Protein Variability Server (http://imed.med.ucm.es/PVS; accessed 02.01.2017) [42]. Secondary structure elements were predicted using the MLRC method [43] at the Network Protein Sequence Analysis WWW server (https://npsa-prabi.ibcp.fr/NPSA/npsa_mlrc.html; accessed 02.01.2017). Phosphorylation sites were predicted by an artificial neural network method [44] at the NetPhos 3.1 server (http://www.cbs.dtu.dk/services/NetPhos/; accessed 02.01.2017).

## Results

### HPgV-1 prevalence in plasma and fetal samples

We took advantage of a large dataset accumulated in the course of routine pathogen screening of plasma and stem cell-containing suspensions which is a part of the standard operational procedures implemented at our Center [33, 38]. In this work, we focus only on suspensions deriving from human fetal tissues (each time collected after elective termination of pregnancy). The term “fetal tissues” means a cell suspension consisting of the liver and some other tissues, depending on preparation conditions and clinical needs. Chorions constitute another subgroup of samples. When analyzed as a single category, chorions and fetal tissues will be hereafter referred to as “fetal material”. In addition, donor’s plasma was routinely collected for diagonostic purposes and tested for pathogens along with fetal samples using commercial real-time PCR kits.

**Fig. 1:**
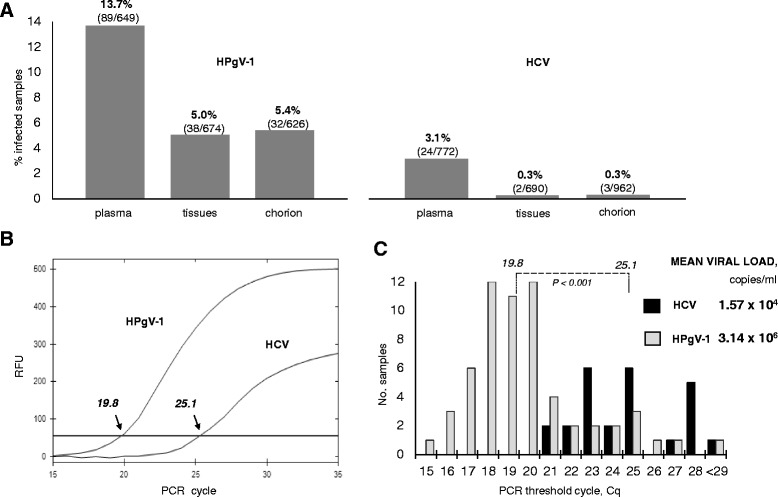
PCR screening of donor plasma and fetal tissues. **a** Percentage of samples of plasma, fetal tissues and chorions infected with HPgV-1 and HCV (as labeled)*.* Absolute counts are given in parentheses below percentages. **b** Kinetics of RT-PCR amplification of HPgV-1 and HCV RNA purified from plasma*.* Shown are representative samples with *Cq* indicated. RFU, relative fluorescence units. **c** Distribution of *Cq* values for HPgV-1- and HCV-infected plasmas (*grey* and *black* histograms, respectively). The differences between mean *Cq* of these PCRs are significant at the level indicated by the *p* value. Mean viral load of HPgV-1 and HCV calculated from corresponding mean *Cq* is shown on the right

The screening revealed HPgV-1 RNA in 13.7% of plasma samples (Fig. 1a) while the prevalence of HPgV-1 in fetal samples was much lower (5.0% and 5.4% of tissues and chorions, respectively). HPgV-1 was detected more frequently than HCV which demonstrated the prevalence of only 3.1% in plasmas and 0.3% in fetal samples. Other routinely tested viruses (HIV, HBV, type 1/2 herpes simplex virus, Epstein-Barr virus, parvovirus B19) were each found in less than 0.01% of samples (data not shown). For comparison, we also provide results of HPgV-1 testing in patients referred to our Center. HPgV-1 and HCV were detected in 4.7% and 0% of the patient plasmas, respectively (*n =* 147).

We next estimated HPgV-1 and HCV load in donor plasmas from the kinetics of PCR amplification. The test kits that we use for routine screening have the similar sensitivity (500 and 380 copies per ml of plasma for HPgV-1 and HCV, respectively). The PCR efficiency is close to 100% as declared by manufacturer and noted in our runs (data not shown). Hence a comparison of PCR template quantities between samples can be drawn from the difference in their *Cq*, a threshold cycle at which the kinetics of amplification passes to the exponential stage (Fig. 1b). The lower *Cq* the higher is the viral RNA content in the sample. For donor plasmas, the mean *Cq* was lower in HPgV-1-detecting PCR (Fig. 1c). The mean viral load, determined from the mean *Cq* (see Materials and Methods), was 3.14 × 10^6^ and 1.57 × 10^4^ for HPgV-1 and HCV, respectively. This data suggests that, in the studied population, HPgV-1 can reach higher blood titers than HCV.

### Dependence of HPgV-1 prevalence in fetal material on donor’s viremia

**Fig. 2:**
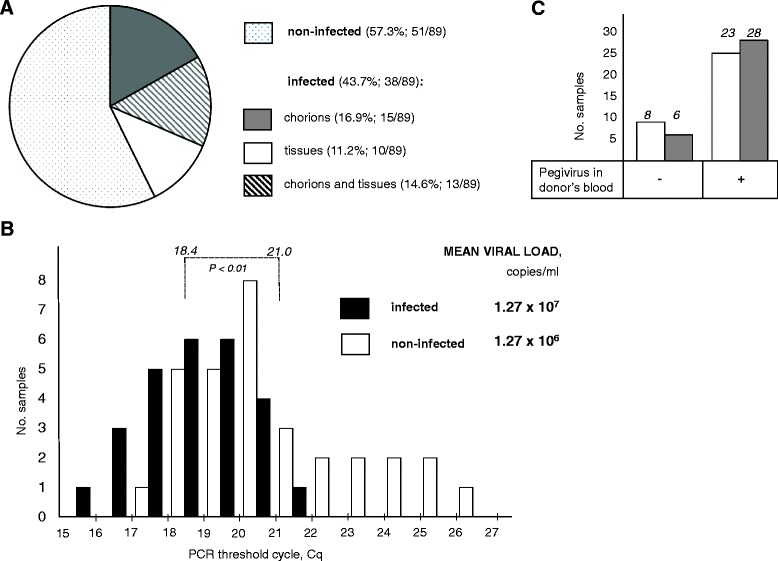
Distribution of HPgV-1 in triads consisting of donor’s plasma, chorion and fetal tissues. **a** Viremic plasmas (total *n = 89*) categorized by the infection status of their cognate fetal tissues and chorions. **b** Dependence of HPgV-1 - infected fetal samples on *Cq* in RT-PCR assay of donor’s plasma. Difference between mean *Cq* for plasma of donors of infected and non-infected fetal material is significant at the level indicated by the *p* value. Mean viral load for the two kinds of plasma is shown on the right. **c** Dependence of the number of infected fetal samples on the presence of pegivirus in donor’s plasma. Total number of triads in each category is shown above hystograms

To further classify HPgV-1 occurrence in preparations used for cell transplantation, we created a dataset of triads consisting of donor’s plasma and samples of donated chorion and fetal tissues. Eighty-nine triads comprising HPgV-1-positive plasma were analyzed (Fig. 2a). Pegiviral RNA was detected in donated fetal samples in 43.7% of these triads. Chorions and tissues were HPgV-1-positive in 16.9% and 11.2% of all triads, respectively, whereas both types of fetal samples contained pegiviral RNA in 14.6%. The higher HPgV-1 RNA content shows that the chorion has a higher capacity to acquire HPgV-1, which could be expected from the anatomy of embryo-maternal contact. It should be noted that the stringency of our sample preparation procedure and the use of mock controls (see Materials and Methods) render unlikely contamination of harvested samples with HPgV-1-infected blood. The observed difference in HPgV-1 prevalence between chorions and tissues further disproves the possibility of lab contamination which would otherwise produce uniform data invariant to the sample type.

Next we asked if the odds to find HPgV-1 in donated tissues depend on the viral level in donor’s plasma. We examined *Cq* values of PCR from HPgV-1-positive plasma samples. For triads with non-infected and infected cognate tissues, mean *Cq* was 21.0 and 18.4 RFU respectively (Fig. 2b). This corresponds to the viral load of 1.27 × 10^6^ of 1.27 × 10^7^ copies/ml, respectively. Therefore, a higher viral load level in plasma increases the chance of finding the virus in fetal samples. In addition to fetal samples which acquired HPgV-1 from viremic donors (infected tissues; Fig. 2b), there was an interesting subset of 15 triads where plasma was pegivirus-negative and at least one fetal sample was nevertheless infected. In this subset, infection was detected in six chorions and eight fetal tissue samples (Fig. 2c, left histograms). These numbers are lower than the corresponding numbers of triads with viremic plasma (28 and 23; Fig. 2c, right histograms) corroborating the significance of the viral load as a predictor of infection of cognate fetal tissues.

To give a numerical estimate of HPgV-1 vertical transmission, we took a rather conservative approach and considered only triads in which the virus was found either in fetal tissues only or both in fetal tissues and chorion. Triads in which only chorion was infected were not included because of the possibility of an admixture of maternal blood. Thus we scored 23 triads of 89 as transmitting which gives the rate of mother-to-fetus transition of 25.8%.

### Nucleotide sequence variability of 5′-UTR and E2 regions

RNA-containing viruses are known to have a potential to generate multiple subtypes (quasispecies) providing rich material for selection of those able to evade host defense mechanisms and cause disease. In pegivirus, variability in the 5′-UTR and E2 regions contributes significantly to the net genotypic diversity [9, 10, 12]. We analyzed portions of these regions by sequencing corresponding PCR products generated from viremic donors. A genotype 2a sequence was used as a reference for the sequence nomenclature. Position 1 is the ATG start codon for the putative polyprotein [6] which corresponds to position 555 in the reference strain D90600. Consequently, the 5′-UTR primer set [39] targets the fragment of −425 to −184 nucleotides (nt), and the E2 set [9] targets the 998-1680 fragment. For 5′-UTR and E2, we obtained 29 and 10 sequences and built the alignments covering −383 to −189 nt and 1035 to 1613 nt, respectively.

The mean pairwise evolutionary distance between references was larger for E2 than 5′-UTR sequences by 0.035 units (Table 1). This agrees with a higher capacity of E2 to support phylogenetic relationships suggested earlier [9]. For samples from HPgV-1-positive donors, we detected an even larger difference in the mean pairwise distances between E2 and 5′-UTR alignments (0.074 units). Accordingly, the mean distance for E2 between samples (0.115) approaches the distance between genotype references (0.140). This might reflect an accelerated accumulation of polymorphisms in the E2 region of HPgV-1 in our samples.

**Table 1:** Mean pairwise evolutionary distances between genotypes and between sequenced samples

Region	Reference genotypes			Samples		
	N	Mean	S.D.	N	Mean	S.D
5′-UTR	7	0.105	0.036	29	0.041	0.030
E2	7	0.140	0.036	10	0.115	0.031
Difference between means		**0.035**			**0.074**	

Sequences of 5′-UTR and E2 regions were analyzed (N, number of sequences). Pairwise distances were estimated separately for two groups of sequences (reference genotypes and samples) with the use of the Tamura-Nei model. Means of distances within each group are given with corresponding standard deviations (S.D.). Differences between the means (bold) for references and isolates are significant at the *p* < 0.001 level (Student’s *t*-test)

**Fig. 3:**
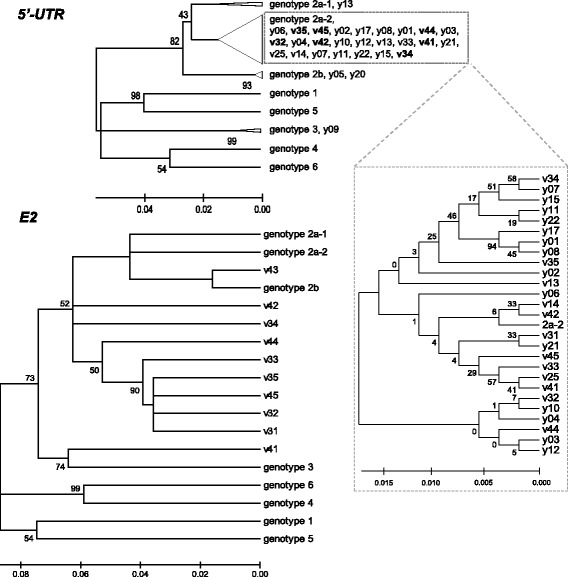
Phylogenetic trees inferred from 5′-UTR and E2 sequences. Neighbor-Joining method was applied to the alignment of sequences of HPgV-1 samples and references: U36380 for genotype 1, D90600 and AF104403 for genotype 2a (2a-1 and 2a-2), U63715 for genotype 2b, AB003288 for genotype 3, AB018667 for genotype 4, AY949771 for genotype 5, and AB003292 for genotype 6. Bootstrap support values higher than 40% are given next to the branches. Bold: samples for which both 5′-UTR and E2 sequences are available. Some sequences were collapsed in groups denoted by triangles. The genotype 2a-2 group is enlarged in (inlet on the right). Scale: evolutionary distances represented as the number of base substitutions per site

To genotype HPgV-1 in viremic donors, we performed the 5′-UTR phylogenetic analysis of the references and 28 of our HPgV-1 isolates (Fig. 3). In the most of these samples (25 of 28), the virus was found to belong to genotype 2a. Genotype 2b was assigned in two samples, and one sample was placed to the genotype 3 group. Note that bootstrap values for revealed clusters were quite high (82% for genotype 2 and 99% for genotype 3), whereas deeper genotyping (2a and 2b) was weaker supported by bootstrapping (43%). Sequences in the genotype 2a subcluster were very similar (evolutionary distances below 0.017) and could no further be classified reliably (Fig. 3; inlet).

It was reported earlier [9, 10, 39] that the portions of E2 and 5′-UTR sequenced in this work are sufficient to reproduce the genotypes delineated using the full-length sequences. Hence we then performed the phylogenetic analysis for the E2 region. Nine of ten E2 sequences were placed to the genotype 2 cluster featuring bootstrap support of only 52%. Inclusion of more references (namely, U44402 and U45966 for genotype 2a and D87709 for genotype 3) did not change the tree topology (data not shown). The genotype 2 cluster contained samples for which both E2 and 5′-UTR sequences were available (bold in Fig. 3). However these samples did not exhibit as strong adherence to genotype 2 in E2 analysis as they did in 5′-UTR analysis. Indeed, the bootstrap values for the genotype 2 clusters were different between the two analyses (82% and 52%, respectively). Moreover, in the E2 tree, branch bifurcation points for samples and references lie in the same range of distances (0.038-0.075; except for isolate v43). This is in contrast to the 5′-UTR tree where bifurcation of references occurs at larger distances than that of samples. It appears that the reliability of E2-based genotyping has been compromised, further arguing for a higher variability of the E2 region of HPgV-1 in donors of fetal material.

Sequencing of 5′-UTR and E2 regions in fetal samples was not successful. The yield of sequencing-grade cDNA was extremely low, probably, due to a low amount of HPgV-1 or/and RNA degradation. We were able to sequence 5′-UTR in only four samples (v34, v35, v45 and y21) and E2 in two samples (v31 and v35). In the portions where alignment could be performed, the sequences were identical to those representing HPgV-1 in cognate maternal plasma (data not shown).

### An elevated variability of the sequenced portion of E2 at the amino acid level

**Fig. 4:**
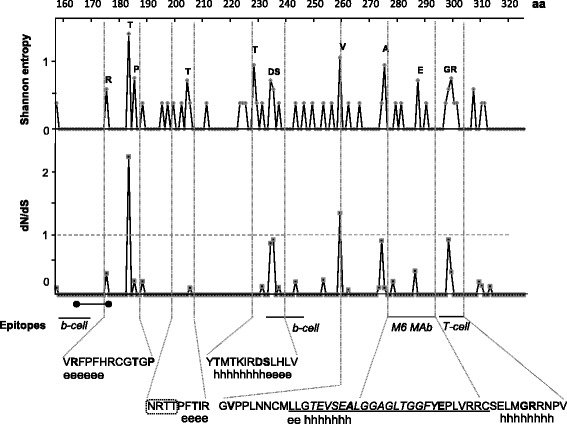
Sequenced fragment of the E2 glycoprotein. Shannon entropy and dN/dS (ordinate) profiles along the translation of the sequenced fragment are presented. Abscissa: amino acid (aa) position in the E2 protein sequence as per genotype 2a reference sequence (GenBank # D90600). Horizontal bars below the plot display epitopes suggested by earlier studies (refs. in text). Highly variable positions are shown in bold in sequences of the context motifs. These amino acids are also shown above the corresponding peaks of the entropy profile. Predicted secondary structure elements are shown under the motif sequences as follows: ee...e – extended strand, hh…h – alpha helix. Membrane fusion peptide is underlined. HIV-inhibitory peptide is italicized. Glycosylation site is circled. Dumbbell-shaped bar indicates palmitoylation site

The E2 glycoprotein carries a series of functional motifs involved in membrane fusion and interaction with components of the immune system [5]. We analyzed the alignment of translations of nucleotide sequence region from 1080 to 1567 nt which corresponds to the polyprotein region of 361 to 537 amino acids (positions 157 to 333 in the E2 glycoprotein [20]). The profile of Shannon entropy, which is one of the ways of visualizing protein sequence variability, contains four peaks reaching the value of 1 (Fig. 4) indicative of a strong variability. A high prevalence of non-synonymous nucleotide substitutions (dn/ds ratio) was detected at five positions that coincide with the entropy peaks. A shift towards higher high dn/ds is usually interpreted as an indication of positive selection going on at the analyzed positions.

Interestingly, the variability peaks are located in putative secondary structure motifs such as polypeptide chain extended strands and alpha helices. These elements in their own turn appear to be associated with a series of antigenic determinants. Examples of this kind are (i) the epitopes for B-cells and CD38+ T-cells predicted by computational analysis [45] and (ii) the epitope for neutralizing murine anti-E2 monoclonal antibodies revealed in an earlier search for immunodominant antigenic sites [18]. Furthermore, the region between two alpha helices (270 - 300) harbors a membrane fusion peptide suggestively playing a key role in the viral entry [46] and, largely overlapping, putative HIV-inhibiting peptide [19]. Other polymorphism-tagged extended strands also appear to be linked to functional elements such as a putative glycosylation site (position 197) [5] and palmitoylation motif (167-175) [45]. Although no role has yet been specified for these elements in HPgV-1 lifecycle, glycosylation and palmitoylation are known to play a role in altering the dynamics of protein-membrane interactions [47].

**Fig. 5:**
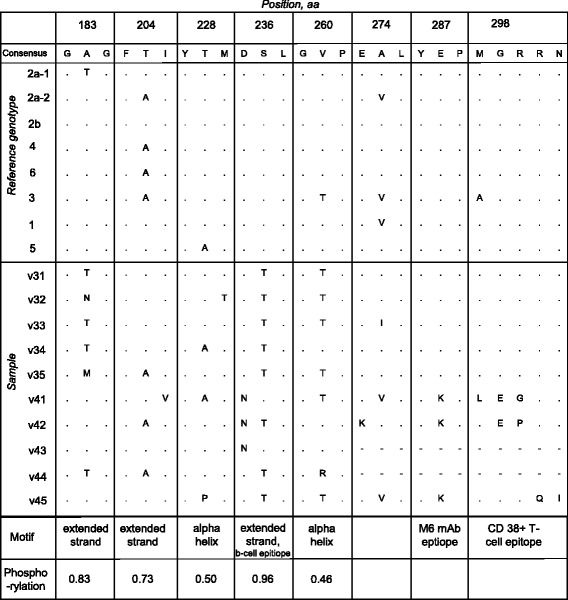
Polymorphisms in E2 characteristic motifs. Blocks containing polymorphic sites highlighted in Fig. 4 are shown. References are the same as in Fig. 3. Positions are as per # D90600 (2a-1). Dots represent identity to the consensus. Dash, sequence is not available. Predicted secondary structure elements and epitopes are shown in the “Motif” row. Phosphorylation potential is given in likelihood units. Values above the threshold (0.5) predict a high probability of the site phoshorylation

We next questioned what sorts of amino acid substitutions are prevalent in the eight selected variable motifs (Fig. 5). Three polymorphisms associated with secondary structure elements add or eliminate a phosphorylation site (positions 183, 204 and 228). Addition of a novel phosphorylation site (although with a low reliability of prediction) is “attempted” at position 260. This change seems to be supported by selection as argued by a peak in the dn/ds profile (Fig. 4). On the other hand, there were two homotypic substitutions (producing no change in the overall amino acid property), Ser236Thr and Ala274Val. These might be considered as evidence of functional and structural indispensability of the corresponding motifs. Remarkably, substitutions at 183, 204, 228, 260 and 274 occur also in genotype references suggesting common driving forces of molecular evolution. In contrast, there were substitutions absent in sequences of selected genotype references. Among these substitutions, noteworthy are Glu287Lys, Gly298Glu and Arg299Pro/Gly drastically changing the amino acid charge. It is remarkable that these substitutions fall in antigenic epitopes, further implying an evolutionary search for novel immunity-evading variants.

## Discussion

Most previous studies on HPgV-1 vertical transmission were based on the detection of the virus in new-born infants [22, 23, 48]. Here we shifted the focus to early-age fetuses and found a high level of pegivirus prevalence (Fig. 1) and transmission (Fig. 2). Our target cohort had an elevated proportion of individuals carrying the virus in plasma (13.7%; Fig. 1a) as compared to two other groups: customers of a commercial medical lab (“The DNA Lab”, Kyiv, Ukraine) and our Center’s patients (8.7% and 4.0%, respectively). Interestingly, this order of decreasing percentages might correlate with the socioeconomic differences in the populations these cohorts likely originate from. It seems logical to assume that women donating aborted fetal material can afford much less medical care than patients receiving expensive stem cell-based treatment at our Center. This agrees with the worldwide prevalence profile for HPgV-1 showing elevated levels in developing countries [5].

The occurence of HPgV-1 was higher in chorions than fetal tissues (Fig. 2a) suggesting gradual transmission. It remains unclear whether HPgV-1 RNA detected by PCR represents a true intracellular infection or results from an uptake of mother blood during earlier embryofetal stages. Also, it is impossible to show that we deal with an infection-competent virus because of a lack of cell culture system suitable for in vitro infection studies. A considerable proportion of infected fetal samples derived from non-viremic donors (Fig. 2c). This presumes the residence of HPgV-1 along the reproductive route, supporting the possibility of infecting the baby during delivery [23, 49]. HPgV-1 persistence at vaginal surfaces seems further plausible given a high frequency of its sexual transmission [50]. Therefore, we conclude that the fetus may acquire the virus not only via blood. Nevertheless the blood level remains the main prognostic factor for HPgV-1 transmission (Fig. 2b) agreeing with the results of earlier perinatal screens [21, 23].

HPgV-1 in most of donors belong to genotype 2 (Fig. 3) which is predominant in Europe [10]. While genotyping by the phylogenetic analysis of the 5′-UTR sequence alignment was quite reliable, sequences of a portion of E2 barely support their grouping with genotype 2 references. This could be due to accelerated evolution in E2 suggested by the analysis of inter-sample and inter-genotypic distances (Table 1). Polymorphisms were found in E2 motifs possessing important structural and functional elements that play a key role in cell binding, HIV inhibition (Figs. 4 and 5) and formation of antigenic landscape. The latter is especially important given attempts of pegivirus to evade the immune pressure [51]. However HPgV-1 seems to balance between the need for a broader E2 antigenic diversification and constraints imposed by the necessity to preserve important functions. This may explain the seeming paradox that pegivirus, reported to be less variable than HCV [7], reaches higher blood titers and rates of vertical transmission (Figs. 1, 2 and previous reports [5]). It was suggested that antibody escape is not the first priority for pegivirus [52]. We speculate that, while HCV tends to accumulate as much E2 variability as possible, the pegiviral strategy may focus more on preservation of E2 natural functions resulting in a broader tropism, particularly towards hematopoetic and immune cells. Indeed, the primary replication sites of HPgV-1 were suggested to localize not in the liver but in the bone marrow and spleen [28]. Establishment of infection in these tissues may require a higher fidelity of functions involved in cell binding, thereby limiting the range of variability in corresponding motifs.

The hypothetic affinity of HPgV-1 to hematopoetic precursors [30] evokes an assumption that contaminated fetal cell suspensions (Fig. 1) may contain this virus in fetal HSC. Therefore, the accidental use of HPgV-1-contaminated cells for transplantation may pose a risk of transporting the virus to the HSC homing sites. Given an inhibiting effect of pegivirus on proliferation (demonstrated recently for differentiating immune cells [51]) the efficacy of HPgV-1-infected stem cells in replacement therapy may be reduced. The consequences may be further aggravated in immunosuppressed patients. Furthermore, we note a higher rate of HPgV-1 detection in comparison to other viruses included in the mandatory test panel (HCV, HIV, HBV, herpex simplex virus, Epstein-Barr virus, parvovirus B19). Therefore, we argue that it would be rather premature to abandon pegivirus PCR testing of samples designed for stem cell therapy despite the fact that such test is not mandatory for blood banks. On the other hand, it looks encouraging to employ pegivirus, benign and capable of gaining high titers, as a vector for HSC-mediated delivery of corrected genetic alleles. This could be put as the basis of widely-discussed strategies for correcting genetic disorders [31, 53] provided all the effects of HPgV-1 in targeted niches are properly evaluated.

## Conclusions

HPgV-1 displays a higher prevalence in donors of fetal stem cell-containing material than HCV. HPgV-1 vertical transmission is quite frequent and could be detected at early development stages. Donor’s blood is the main, but not the only, source of fetal infection. Most of HPgV-1 isolates belong to genotype 2 as could be determined by sequence analysis of 5′-UTR. Sequencing of the E2 glycoprotein in a smaller subset of samples placed them to genotype 2 as well, but with a lower reliability. This could result from accelerated accumulation of variability in E2 exceeding the extent optimal for reliable genotyping. Polymorphisms often occur in E2 motifs bearing structural, functional and immunogenic importance. An ongoing selection for better-fit variants could be implied. This makes difficult to predict the effect of HPgV-1 persistence in host cells, particularly HSC believed to be the primary site of HPgV-1 replication. Given the wide therapeutic use of fetal stem cells (represented largely by HSC), we advocate the necessity of HPgV-1 testing of fetal material and donors thereof.

## Abbreviations

*Cq*: The threshold cycle of PCR amplification
HPgV-1: Human pegivirus
HSC: Hematopoetic stem cells
PCR: Polymerase chain reaction
RFU: Relative fluorescent units
